# Dietary supplements consumption and its association with socioeconomic factors, obesity and main non-communicable chronic diseases in the north of Iran: the PERSIAN Guilan Cohort Study (PGCS)

**DOI:** 10.1186/s40795-021-00488-2

**Published:** 2021-12-15

**Authors:** Marjan Mahdavi-Roshan, Arezoo Rezazadeh, Farahnaz Joukar, Yasaman Khorshidi, Mohammadreza Naghipour, Fariborz Mansour-Ghanaei

**Affiliations:** 1 Cardiovascular Diseases Research Center, Department of Cardiology, Heshmat Hospital, School of Medicine, Guilan University of Medical Sciences, Rasht, Iran; 2 Gastrointestinal and Liver Diseases Research Center, Guilan University of Medical Sciences, Razi Hospital, Sardar-Jangle Avenue, Rasht, Iran; 3 Department of Community Nutrition, National Nutrition and Food Technology Research Institute, Faculty of Nutrition Sciences and Food Technology, Shahid Behehshti University of Medical Sciences, Tehran, Iran; 4 Caspian Digestive Diseases Research Center, Guilan University of Medical Sciences, Rasht, Iran; 5 GI Cancer Screening and Prevention Research Center, Guilan University of Medical Sciences, Rasht, Iran

**Keywords:** Dietary supplements, Non-communicable diseases, Hypertension, Diabetes, Obesity, PERSIAN Guilan cohort study, Socioeconomic factors

## Abstract

**Background:**

Dietary supplements (DSs) use have become a growing trend worldwide, and it may be affected by demographic and sociocultural factors. Some people use supplements with the thought that they can improve their health, reduce symptoms and prevent disease. The aim of the present study was to define the frequency of DS use and its association with socioeconomic factors among participants with selected main non-communicable chronic diseases (NCDs) (diabetes, cardiovascular disease (CVD), hypertension (HTN), cancers, and obesity in the north of Iran.

**Methods:**

This large cross-sectional study was conducted as a part of the PERSIAN Guilan cohort study. Supplement use during last year and its type, demographic factors, socioeconomic status, lifestyle habits were asked by face-to-face interview. The history of chronic disease was defined by a trained team. Data were analyzed using SPSS. The chance of supplement use according to demographic, socioeconomic, and lifestyle variables and history of chronic disease was analyzed by logistic regression.

**Results:**

10,520 men and women aged 35–70 years in Some’e Sara County (including urban regions and 39 villages) were studied. About 25% of participants consumed DSs. The highest consumption of DS was calcium/vitamin D (11.1%), ferrous sulfate (8.8%), and vitamin D pearl or ampoule (7.7%). The highest percent of the history of chronic disease was central obesity (62.7%), HTN (43.2%), and general obesity (32.7), respectively. After adjustment for confounders, those with female gender, the highest age ranges (55–65 and > 65 years), high academic education, living in urban regions, and good economic status were more likely to be DSs consumers; however, married and smoker subjects were more likely to consume DS. Participants who had a history of diabetes, HTN, CVD, Obesity, and Central Obesity were more likely to intake DS in comparison with healthy subjects.

**Conclusion:**

This study showed that a quarter of the participants were DS users. Female sex, older age groups, and higher educated participants, and among chronic disease, patients with HTN, CVD, and diabetes were more likely to be users of any DS.

**Supplementary Information:**

The online version contains supplementary material available at 10.1186/s40795-021-00488-2.

## Background

Following the epidemiological transition, non-communicable diseases (NCDs) are a major part of the community’s health problems [1]. According to the definition of the World Health Organization (WHO), the main types of NCDs are cardiovascular diseases (CVD), cancers, chronic respiratory diseases, and diabetes which are collectively responsible for almost 70% of all deaths worldwide [2]. This organization also predicts that NCDs will be responsible for three-quarters of all deaths in the world by 2030 [3]. In Iran, the first rank of death is attributed to cardiovascular disease, and the third is cancer [4–7]. Many factors may contribute to the development of chronic diseases, including genetics and environmental factors [8–10]. The rising trend of obesity has also made it one of the main leading causes of NCDs in the world [11–13], which makes it the important risk factor of other chronic diseases such as cardiovascular diseases and cancers [14].

Previous studies showed that adults with cancer or other chronic diseases tend to use supplements more than healthy subjects [15, 16], and according to NHANES data, dietary supplements use has been rising since the 1970s [17]. Based on the Food and Drug Administration description, a dietary supplement is a product that contains nutrients to increase the nutritional value of one’s diet. Using dietary supplements such as vitamins, antioxidants, fiber, trace elements, amino acids has become an important health trend worldwide [18]. Nowadays, various types of dietary supplements are widely sold through pharmacies [19, 20]. It is estimated that more than 50% of adults in the UK use at least one dietary supplement daily [21]. Some studies have suggested the role of supplements in preventing or progression of chronic diseases [22, 23]; however other studies have not found a positive association between supplementation and chronic diseases [24–26]. Also, some health experts are concerned about the interactions of these supplements with the drugs used by people with chronic diseases, especially those with cancer [16]. Nevertheless, most people use supplements with the thought that supplements are natural and safe compounds that can improve their health, reduce symptoms and prevent disease [27].

The use of dietary supplements has become a growing trend worldwide, and it may be affected by demographic and sociocultural factors. Still, studies about the use of dietary supplements in Iran are limited, and there is not enough information about dietary supplement consumption status in the north of Iran. To the best of our knowledge, this is the first study to investigate the consumption of commonly available dietary supplements and their association with socioeconomic factors in participants with selected main NCDs (diabetes, CVD, hypertension (HTN), cancers, obesity, and central adiposity) in the north of Iran.

## Materials and methods

### Subjects

The present cross-sectional study was conducted within the framework of the PGCS; a study performed on 10,520 men and women between 35 and 70 years old in Some’e Sara County (including urban regions and 39 villages) that is located in northern Iran, from October 8, 2014 to January 20, 2017 as part of the Prospective Epidemiological Research Studies in Iran (PERSIAN) [28, 29].

Men and women in the age range of 35–70 residing in urban and rural areas of Some’e Sara county located in North of Iran who used dietary supplementations were included in the present study. Participants were excluded if they were unable to attend the clinic for interview and physical examination, if they had mental retardation, or if they were unwilling to participate.

Data were obtained by a questionnaire by a face-to-face interview with trained interviewers. Regarding supplement use, people were asked if they were regularly using even one of the dietary supplements (DS) (Mineral Multivitamin, Multivitamin, Calcium-D, Calcium, Vitamin D, Folic acid, ferrous sulfate, zinc sulfate, and omega-3) during last year and its type. Participant characteristics, including demographic (age, gender, family number) characteristics, socioeconomic status (educational status, occupation, household economic status), lifestyle habits (smoking and alcohol consumption habit), employment status, and anthropometric indices including weight (kg), height, etc. were measured by trained health care providers. Body mass index (BMI) was calculated as weight (in kg) divided by height squared (in m^2^). Obesity and central obesity were defined as BMI ≥30 kg/m2 and waist ≥95 cm [based on national cut-off] [30], respectively. History of chronic illnesses including diabetes, HTN, CVD, and cancers was assessed by sophisticated physicians of the team. Diabetes was defined as fasting blood glucose equal to or higher than 126 mmol/L, or was on medication for raised blood glucose, or had a history with the diagnosis of diabetes [31]. HTN was defined as systolic blood pressure (SBP) ≥130 mmHg, and /or a diastolic blood pressure (DBP) ≥80 mmHg according to the ACC/AHA guideline, a prior diagnosis of hypertension by a health professional indicating that one had high BP or used antihypertensive drugs [32]. CVD includes the history of myocardial infarction or/and stroke or/and cardiac ischemia. Alcohol consumption and smoking habits were asked by yes or no question.

Validation of other measurements such as blood pressure instruments has been performed in previous studies [33–36].

#### Statistical analysis

Data were analyzed using the statistical software package SPSS Version 16.0. Participants’ age was divided into four age groups: 35 to < 45 years, 45 to < 55 years, 55 to < 65 years, and 65 years and over. Household economic status was defined by scoring owning house status and its room number and infrastructure per capita and household assets, based on their economic value according to a previous study [28], and computing the total score. The total score was categorized into tertiles (T), and T1, T2, and T3 were labeled as low, middle, and high SES, respectively.

To compare the general characteristics of participants between two living regions (urban and rural), χ2 tests were used. The chance (95% confidence interval [95%CI]) of supplement use according to demographic, socioeconomic, and lifestyle variables and history of chronic disease was analyzed by logistic regression simultaneously adjusted for all assessed variables (demographic, socioeconomic, lifestyle factors). In the regression models, the supplement use status was considered as a dependent binary variable. *P* < 0·05 was considered as significant.

## Results

Table 1 represents the sociodemographic characteristics, history of NCDs, and the supplement use status of the participants (*n* = 10,520), of which 56.2% (*n* = 5907) and 43.8% (*n* = 4613) lived in rural and urban regions, respectively. Most of them were 45 ≤ or < 55 years old (36.6%). Among total participants, the majority of the respondents aged < 55 years (66.5%), married (90.6%). Half of them were illiterate or had under 6 years of education (48%). A quarter of individuals consumed DS and 14% of them had alcohol consumption habits. The highest consumption of DS reported by participants was calcium/vitamin D (11.2%), ferrous sulfate (8.8%), and vitamin D pearl or ampoule (7.7%). The highest percent of the history of chronic disease was central obesity (62.7%), HTN (43.2%), and obesity (32.7%), respectively. Demographic, socioeconomic, and lifestyle characteristics of participants based on regions of living varied significantly. Based on the regions, individuals who lived in rural areas had higher consumption of DSs than those residing in urban regions. Furthermore, the frequency of diabetes and hypertension were significantly higher in urban areas.

**Table 1:** General characteristics of the study population according to living region

Region			Total N(%)	*P*^1^
	Rural N(%)	Urban N(%)		
Gender,				
Female	3082 (52.2)	2551 (55.3)	5633 (53.5)	0.001
Male	2825 (47.8)	2062 (44.7)	4887 (46.5)	
Age (years)				
35- < 45	1792 (30.3)	1350 (29.3)	3142 (29.9)	0.000
45- < 55	2202 (37.3)	1650 (35.8)	3852 (36.6)	
55- < 65	1567 (26.5)	1163 (25.2)	2730 (26.0)	
≥ 65	346 (5.9)	450 (9.8)	796 (7.6)	
Educational status				
Illiterate	729 (12.3)	1009 (21.9)	1738 (16.5)	0.000
1–5 years of schooling	1577 (26.7)	1735 (37.6)	3312 (31.5)	
6–12 years of schooling	3074 (52.0)	1758 (38.1)	4832 (45.9)	
University/college	527 (8.9)	111 (2.4)	638 (6.1)	
Marital status				
Married	5393 (91.3)	4134 (89.6)	9527 (90.6)	0.003
Single/Widowed/Divorced	514 (8.7)	479 (10.4)	993 (9.4)	
Current cigarette smoking, No. (%)				
Yes (daily)	895 (15.2)	738 (16.0)	1633 (15.5)	0.403
Yes (sometimes)	114 (1.9)	80 (1.7)	194 (1.8)	
no	4446 (75.3)	3420 (74.1)	7866 (74.8)	
Past smoking	452 (7.7)	375 (8.1)	827 (7.9)	
Drinking Alcohol (yes)	905 (15.3)	610 (13.2)	1515 (14.4)	0.002
History of chronic disease				
Diabetes	1364 (23.1)	1167 (25.3)	2531 (24.1)	0.009
Hypertension	2357 (39.9)	2189 (47.4)	4543 (43.2)	0.000
Cardiovascular disease	457 (7.7)	403 (8.7)	860 (8.2)	0.063
Cancer	60 (0.6)	34 (0.3)	94 (0.9)	0.132
Obesity	1970 (33.4)	1466 (31.8)	3436 (32.7)	0.08
Central Obesity	3738 (63.3)	2856)61.9(	6594 (62.7)	0.15
Dietary Supplement usage	1611 (27.3)	991 (21.5)	2602 (24.7)	0.000
Type of Supplement				
Calcium	44 (0.7)	30 (0.7)	74 (0.7)	0.565
Multivitamin/mineral	34 (0.6)	23 (0.5)	57 (0.5)	0.593
Multivitamin	69 (1.2)	58 (1.3)	127 (1.2)	0.678
Calcium/vitamin D	737 (12.5)	443 (9.6)	1180 (11.2)	0.000
Vitamin D pearl/ampoule	574 (9.7)	240 (5.2)	814 (7.7)	0.000
Folic acid	443 (7.5)	268 (5.8)	711 (6.8)	0.001
Omega 3	46 (0.8)	24 (0.5)	70 (0.7)	0.106
Ferrous sulfate	548 (9.3)	383 (8.3)	931 (8.8)	0.810
Zinc sulfate/Zinc gluconate	28 (0.5)	13 (0.3)	41 (0.4)	0.116

^1^*P*-values were calculated by Pearson’s χ2 test where appropriate

Table 2 represents the characteristics and history of NCDs in dietary supplement consuming participants across living regions. In total participants, a higher percentage of DS users were women, married, in the age range of 45 ≤ age < 55 years, had higher educational years, not having alcohol consumption habits. Among DS consumers, a higher percentage of those with a history of HTN lived in urban regions.

**Table 2:** Characteristics of dietary supplement consuming participants (*N* = 2602) across living regions

Characteristics	Rural (*N* = 1611) N (%)^1^	Urban (*N* = 991) N (%)^1^	Total (*N* = 2602) N (%)^1^	*P*^2^
Gender				
Female	1347 (13)	879 (8)	2226 (21)	< 0.001
Male	264 (2.5)	112 (1)	374 (3.5)	
Age (years)				
35- < 45	471 (29.2)	256 (25.8)	727 (27.9)	< 0.001
45- < 55	594 (36.9)	349 (35.2)	943 (36.2)	
55- < 65	458 (28.4)	270 (27.2)	728 (28.0)	
≥ 65	88 (5.5)	116 (11.7)	204 (7.8)	
Educational status				
Illiterate	213 (13.2)	239 (24.1)	452 (17.4)	< 0.001
1–5 years of schooling	396 (24.6)	335 (35.8)	751 (28.9)	
6–12 years of schooling	832 (51.6)	366 (36.9)	1198 (46)	
University/college	170 (10.6)	31 (3.1)	201 (7.7)	
Use alcohol				
Yes	72 (4.5)	38 (3.8)	110 (4.2)	< 0.001
No	1539 (95.5)	953 (96.2)	2492 (24)	< 0.001
Current cigarette smoking, No. (%)				
Yes (daily)	234 (17.1)	201 (16.3)	435 (16.7)	0.37
Yes (sometimes)	23 (1.7)	15 (1.2)	38 (1.5)	
no	1023 (74.7)	917 (74.4)	1940 (74.6)	
Past smoking	90 (6.6)	99 (8.0)	189 (7.3)	
Marital status				
Single/Widowed/ Divorced	193 (12.0)	144 (14.5)	337 (13.0)	0.06
Married	1418 (88.0)	847 (85.5)	2265 (87.0)	
Having history of non-communicable diseases				
Cardiovascular disease	110 (8)	107 (8.7)	217 (8.3)	0.063
Diabetes	310 (22.6)	312 (25.3)	622 (23.9)	0.1
Hypertension	543 (39.6)	598 (48.5)	1141 (43.9)	0.000
Cancer	60 (0.6)	34 (0.3)	94 (0.9)	0.132
Obesity	464 (33.9)	397 (32.2)	861 (33.1)	0.37
Central Obesity	874 (63.8)	779 (63.2)	1653 (63.5)	0.76

^1^Number (percent)

^2^*P*-values were calculated by Pearson’s χ2 test where appropriate

The odds ratio (95% Confidence Interval) of dietary supplement use according to socioeconomic characteristics and history of chronic disease of participants was shown in Tables 3 and 4. After adjustment for all variables in the model, female in comparison to male, those at the highest age ranges (55 ≤ age < 65, and ≥ 65 years) in comparison with those at the lowest age range (35-45 years), Post Graduates in comparison with individuals with 0–11 years education, those living in urban regions in comparison with rural areas, participants with medium and high economic status in comparison with those with Low economic status were more likely to consume DS. In contrast, married and smoker subjects were less likely to use DS (Table 3). Participants with diabetes, HTN, CVD, Obesity, and Central Obesity appeared to be more likely to consume DS in comparison with healthy subjects. After adjustment for confounders (demographic, socioeconomic, and lifestyle), the significance remained for diabetes, HTN, and CVD (Table 4).

**Table 3:** Odds ratio (95% Confidence Interval) of supplement use according socioeconomic characteristics of participants^1^

	OR (95%CI)	*P*-value	P-trend^1^
Gender			< 0.001
Female	6.59 (5.50–7.91)***	< 0.001	
Male	Ref. (1)		
Age			< 0.001
35- < 45	Ref. (1)		
45- < 55	1.12 (0.99–1.27)	0.06	
55- < 65	1.39 (1.19–1.63)***	< 0.001	
≤65	1.68 (1.21–2.34)**	< 0.01	
Living region			< 0.001
Urban	1.49 (1.12–1.92)***	< 0.001	
Rural	Ref. (1)		
Marital status			
Married	0.99 (0.81–1.12)	0.93	0.08
Single/Widowed/Divorced	Ref. (1)		
Educational status			< 0.001
Illiterate	Ref. (1)		
1–5 years of schooling	0.74 (0.57–0.95)*	< 0.05	
6–12 years of schooling	1.19 (1.04–1.37)*	< 0.05	
Smoking (yes &sometimes)^3^	0.73 (0.58–091)**	< 0.01	
Drinking Alcohol (yes)^3^	0.99 (0.76–1.27)	0.94	
Economic status			< 0.01
Low	Ref. (1)		
Medium	1.18 (1.03–1.35)*	< 0.05	
High	1.28 (1.11–1.46)***	< 0.001	

^1^Each variable in the model was adjusted for the effect of the other variables

^2^*P* values and P-for trend were calculated by logistic regression analysis

^3 “^No” considered as the reference group

*p < 0.05, ** *p* < 0.01, ***p < 0.001

**Table 4:** Odds ratio (95% Confidence Interval) of dietary supplement use according to participants^a,b^

	Crude Model	Adjusted Model^a^
History of chronic disease	**OR (95%CI)**	**OR (95%CI)**
Diabetes	1.34 (1.21–1.48) ***	1.18 (1.04–1.34)**
Hypertension	1.28 (1.17–1.39) **	1.24 (1.11–1.38 (**
Cardiovascular disease	1.33 (1.14–1.55) ***	1.19 (1.08–1.61)*
Cancer	0.85 (0.73–1.00)	0.92 (0.76–1.21)
Obesity	1.65 (1.50–1.80) ***	1.05 (0.92–1.18)
Central Obesity	1.71 (1.56–1.89) ***	1.09 (0.98–1.23)

^a^adjusted for age, gender, living region, educational status, economic status, smoking and alcohol consumption habits

^b^The supplement use was considered as dependent binary variable, and the history of diseases are considered as exposures in the regression model

*p < 0.05, ** *p* < 0.01, ***p < 0.001 (*P* value was calculated by logistic regression analysis)

## Discussion

This large cross-sectional study assessed the status of DSs use in participants with selected main NCDs (diabetes, CVD, HTN, cancers) and obesity and central adiposity as well as the sociodemographic characteristics of supplement users in the north of Iran.

Findings showed a quarter of our population consumed DS and the highest reported DS was calcium/vitamin D. Data from the *United States* national Health and Nutrition Examination Survey in 2017–2018 showed that 57% of adults have taken dietary supplements in the last 30 days and Supplements that were common in all age groups were multivitamins, followed by vitamins D and omega-3. These data also show an increasing trend in supplement consumption over the past decade [37]. Evidence about DS use in the world shows that, in 2015, 71% of the Danish population were dietary supplement users [38]. Reportedly, in 2017–2018 more than 50% of adults residing in the United States use DS on a regular basis for a variety of health reasons [39, 40]. Also, based on an Australian health survey during 2011–2012, less than one-third of Australians used a dietary supplement [41]. In Iran, a study in 2010 found that 41.9% of health centers visitors in west Tehran had used at least one type of dietary supplement [42]. In our population, DS use was associated with sex, age, educational level, urban living region, and economic status, while married participants were more likely to consume DS. These results are consistent with the findings from other studies. In a study [43] on 1633 students and staff members of a university in Australia, age, sex, and income factors were associated with the use of dietary supplements during illness. In a narrative review, Felicity et al. [41] showed that applying complementary and alternative medicine was related to the demographic characteristics of users. Some studies have also shown that supplement users are more likely to be female, old, and have a higher educational level [41, 43–47].

In our study, there was a significant difference between men and women in consuming DS. These differences may partly reflect marketing strategies targeting females. Alternatively, men and women may have different perceptions of health behaviors as well as nutrition and lifestyle, and women have a higher tendency to use supplements in order to prevent disease [48]. Although some studies have identified marital status associated with DS use [45, 49], in our population, it did not seem to be a significant predictor.

Based on our results, the most DSs use was observed among those with academic education. Previous studies have shown that people with higher literacy have been reported to be more likely to intake DSs [44, 45], probably due to strong awareness of eating needs.

In the present study, non-smokers and those who did not drink alcohol were more likely to use DS, which was consistent with findings of previous studies [38, 50]. A cross-sectional study on 54,948 Danish people, aged 50 ≤ and < 64 years, showed that people with healthy lifestyles and more health-related awareness were more likely to be a DS users [38].

There is not sufficient evidence for reasons of higher consumption of DS in NCD patients of the studied population. It is assumed that patients may incline to change their life-style and to adopt some healthier and more protective habits such as healthy dietary habits and DS consumption following their doctors advice for controlling their disease [51].

Participants with CVD and those suffering from hypertension were more likely to consume DS in our study. In a meta-analysis, Bin et al. reviewed 20 studies that investigated the prevalence of DS use in cardiac patients in Australia. The result of the study revealed that overall, 36% of cardiac patients use DS [52]. Another study by Karny-Rahkovich et al. in 2015 evaluated DS consumption in patients with cardiac disease; this study showed that DS use is common in cardiac patients, and 45% of them consumed DS [53]. Gohar et al., in a Cross-sectional questionnaire survey in 2008 on hypertensive patients in the UK, showed that the prevalence of complementary and alternative medicine use was higher in hypertensive patients than in the UK population, and the most commonly used CAM (complementary and alternative medicine) were vitamins [54]. CAM often was chosen by consumers instead of or in addition to antihypertensive medications [55]. Data of Epirus Health cohort study showed that women with chronic diseases are more likely to receive supplements, and also in this data DS use showed an inverse association with diastolic blood pressure in women [56].

Our results showed that nearly 30% of diabetic participants consumed DS. This result was approximately one-third of the frequency of consumption reported by Ewers et al. in 2018 in the Danish population which reported almost all (99%) of people with diabetes were taking dietary supplements [57]. On the other hand, a recent study found that DS use was lower in people with a diagnosis of diabetes [58].

In our population, obese participants and those with central obesity were more likely to consume DS in comparison with healthy subjects. This result is inconsistent with previous studies. Some studies have found that overweight/obese individuals are less likely to use supplements [38, 59, 60]. Barnes et al. showed that DS use is higher in people with BMI less than 24.9 kg/m^2^ [43]. Also, findings of Burnett et al. on Australian adults from the 2011–2012 National Nutrition and Physical Activity Survey did not find a relationship between BMI and supplement use [41].

Previous studies showed that DS use is common among cancer survivors [61–63]. Friedman et al. found that participants with cancer were more likely to use DS than those whithout cancer, which after adjusting for age, race and gender, the difference between groups *was no longer significant* [58]*.* In our population, only about 1% of cancer-diagnosed patients consumed DS. This may be because studies exploring the effect of DS on cancer outcomes are mixed, with some studies reporting benefits and others finding significant adverse outcomes [39]. Also, the prevalence of cancer was very low in our study population; hence, data was limited to draw any conclusion. There is also a concern that DS might interfere with cancer treatments. For this reason, oncologists often recommend that patients avoid dietary supplements until their cancer treatment is over and encouraged to obtain micronutrients and phytonutrients associated with a reduced risk of cancer by consuming a diet rich in fruits, vegetables, legumes, and nuts [47].

There are some strengths and limitations to consider when interpreting the results of this study. A large sample size and considering geographic distribution from both rural and urban residents increases the accuracy and generalizability of results to the whole population. Some limitations of this study include a cross-sectional design that did not let us investigate any causal relationships. Also, self-report of DSs use may mask the actual intake report.

## Conclusion

In conclusion, this study showed that a quarter of the participants were users of dietary supplements. Females, older age groups, and higher educated participants, and regarding NCDs, patients with HTN, CVD, and diabetes appeared to be more likely to consume dietary supplements.

## Supplementary Information

**Additional file 1:** **Fig. 1.** Geographical location using Garmin GPS MAP78s of the dietary supplements (DSs) users and non-users (The map depicted in figure is our own work).

## Data Availability

The study protocol and the datasets analyzed are available from the corresponding author upon request.

## Abbreviations

DSs: Dietary supplements
NCDs: Non-communicable chronic diseases
CVD: Cardiovascular disease
HTN: Hypertension
WHO: World Health Organization
BMI: Body mass index
SBP: Systolic Blood Pressure
DBP: Diastolic Blood Pressure
T: Tertile
CAM: Complementary and alternative medicine
