# Supplementary material for: Dietary supplements consumption and its association with socioeconomic factors, obesity and main non-communicable chronic diseases in the north of Iran: the PERSIAN Guilan Cohort Study (PGCS)
Source: BMC Nutr. 2021 Dec 15;7:84. doi: 10.1186/s40795-021-00488-2 (PMC8672625; doi:10.1186/s40795-021-00488-2)
Supplement: Supplementary file 1 — Additional file 1: Fig. 1. Geographical location using Garmin GPS MAP78s of the dietary supplements (DSs) users and non-users (The map depicted in figure is our own work). [file 40795_2021_488_MOESM1_ESM.docx]

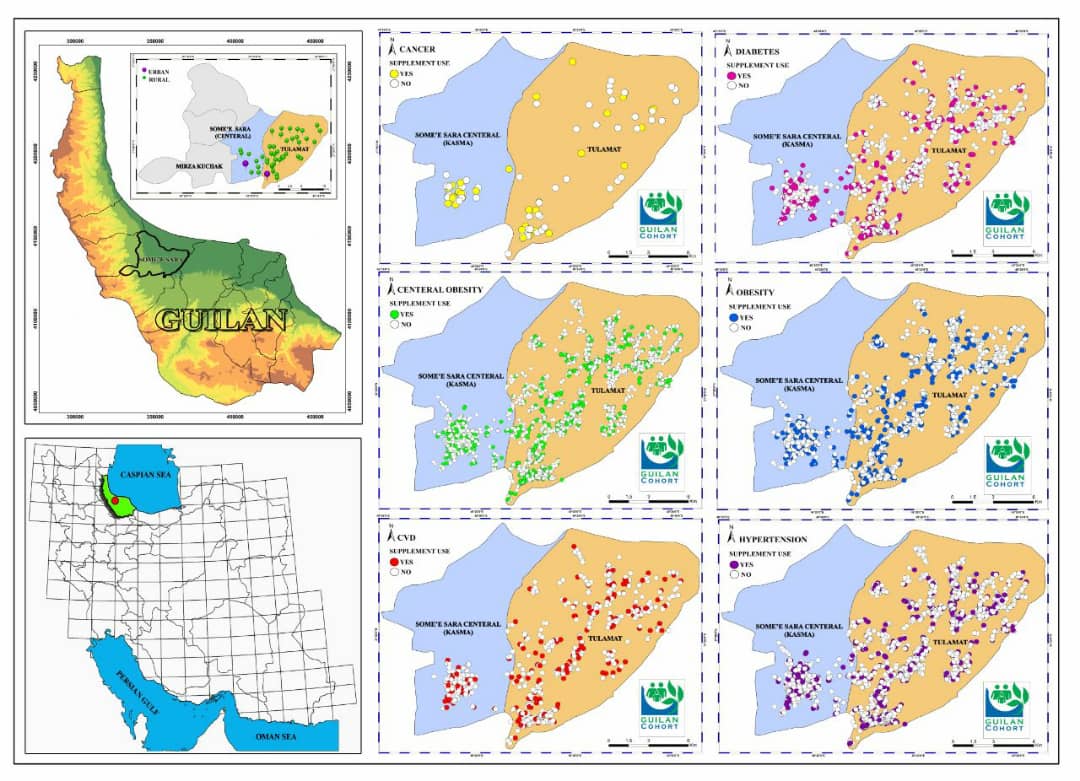


Fig 1. Geographical location using Garmin GPS MAP78s of the dietary supplements (DSs) users and non-users (The map depicted in figure is our own work)
